# Less Is More: Ulnar Lengthening Alone without Radial Corrective Osteotomy in Forearm Deformity Secondary to Hereditary Multiple Exostoses

**DOI:** 10.3390/jcm8111765

**Published:** 2019-10-23

**Authors:** Po-Jen Hsu, Kuan-Wen Wu, Chia-Che Lee, Ken N. Kuo, Jia-Feng Chang, Ting-Ming Wang

**Affiliations:** 1 Department of Orthopedic Surgery, National Taiwan University Hospital, Taipei City 100, Taiwan; 2 Cochrane Taiwan, Taipei Medical University, Taipei City 110, Taiwan; 3 Department of Internal Medicine, Shuang Ho Hospital, Taipei Medical University, New Taipei City 235, Taiwan

**Keywords:** hereditary multiple exostoses, forearm deformities, ulnar lengthening, radial corrective osteotomy, radial head dislocation

## Abstract

Ulnar lengthening has gained popularity in treating forearm deformity due to hereditary multiple exostoses (HME). Whether a simultaneous radius angular correction is necessary for bowing deformity remains debatable. We aimed to evaluate effectiveness and safety of ulnar lengthening alone in HME children. HME patients with forearm deformity who underwent ulnar lengthening between 2011 and 2016 were included. Patients were divided into two groups: eight juniors (age ≤ 10 years) and six seniors (>10 years). The mean age of two groups was 8.1 ± 2.5 and 16.7 ± 4.4 years, respectively. The juniors underwent ulnar lengthening alone, and the seniors received an additional radial corrective osteotomy. Pre-operative and post-operative parameters of supination, pronation, ulnar variance (UV), radial articular angles (RAA), and carpal slip (CS) were assessed. The juniors rather the seniors had an improvement in supination (*p* < 0.05 and *p* = 0.109, respectively). The juniors and seniors improved in pronation (*p* < 0.05). UV, RAA, and CS were corrected in the seniors (*p* < 0.05). In the juniors, parameters improved in UV, RAA, and CS (*p* < 0.05). For HME children, ulnar lengthening alone can restore radiologic anatomy and functions, providing comparable surgical outcomes in cosmetic results and clinical parameters.

## 1. Introduction

Hereditary multiple exostoses (HME), an autosomal dominant disease, is associated with loss-of-function mutations of the EXT1 and EXT2 genes [1]. It is characterized by the growth of benign, cartilage-capped osseous tumors called osteochondromas (or exostoses) over the long bones and axial skeleton. These osteochondromas, usually forming adjacent to the physis of the bones, can cause growth plate disturbances and growth deformity. Patients with HME commonly suffer pain, limited joint motions, and cosmetic problems, which adversely impacts their quality of life [2,3]. HME children also participated less in sport and physical activity than general population in the same age [4].

About 30–60% of HME patients have forearm deformities [5,6], and relative shortening of the ulna is the most typical deformity. The greater contribution to longitudinal growth and the smaller cross-sectional area of the distal ulnar physis as compared to the radius could explain the vulnerability of growth affected by osteochondromas [6]. Therefore, ulnar shortening is usually accompanied by bowing of the radius to compensate radio-ulnar length discrepancy, resulting in ulnar translocation of the carpus, angular deviation of the distal radial epiphysis and possible radial head dislocation.

Many surgical procedures have been employed to treat the forearm deformities in HME patients [7,8,9]. Currently, ulnar lengthening is the most prevalent procedure [10]. Lengthening is conducted with either one stage method or gradual method. One stage lengthening method applied to patients with a small discrepancy usually requires an osteotomy and immediate lengthening with bone grafting [11]. Gradual lengthening is conducted through progressive callus distraction using an external fixator. Additionally, a combination of other procedures along with ulnar lengthening is commonly performed, such as excision of osteochondromas and radial corrective osteotomy [10]. However, there is no consensus regarding the surgical modalities nor timing of surgical intervention.

In this single institutional study, we reviewed the outcomes of gradual ulnar lengthening for ulnar shortening deformities in HME patients who underwent consistent procedures. We aimed to evaluate the necessity of corrective osteotomy for radial angular deformity in skeletally immature patients. Our hypothesis is that the juniors (age ≤ 10 years) have the potential of remodeling to restore the forearm function without radial corrective osteotomy.

## 2. Materials and Methods

We retrospectively reviewed the medical records of patients with HME and forearm deformities following surgical procedures at our hospital. Because HME is a rare condition, we included all HME patients who underwent ulnar lengthening procedures between 2011 and 2016 in the study without loss of follow-up. Fourteen patients (5 female and 9 male) underwent gradual ulnar lengthening using a monolateral external fixator between 2011 and 2016. Conservative managements have been recommended for pediatric patients with forearm fractures or plastic deformation, especially in children less than 10 years old [12,13]. Thus our patients were further divided into two groups: the senior group (age > 10 years) comprised six patients who underwent a combination of ulnar lengthening and radial corrective osteotomy. The junior group (age ≤ 10 years) consisted of eight patients with ulnar lengthening alone. A radial corrective osteotomy was not performed on skeletally immature patients. All surgical procedures were performed by the same surgeon (T.M. Wang).

Surgical indications included intolerable pain during motion, limited forearm range of motion that affected daily activities, and impending radial head dislocation. We simultaneously performed excision of osteochondromas if the tumor was either seen near the physis, palpable as a protruding mass with tenderness or interfered with joint motion. If indicated, a concurrent radial correction was performed by a closing wedge osteotomy at the center of rotation of angulation (CORA) of the radius with plate fixation. For ulnar lengthening, if the patient had preoperative radial head dislocation or impending dislocation, the ulnar osteotomy was performed at the proximal third of the ulna for radial head reduction. If there was an ulnar deformity, the osteotomy was performed at the CORA of the ulna followed by an immediate angular correction. Otherwise, we performed the osteotomy at the distal third of ulna. Subsequently, the monolateral external fixator (Orthofix, Bussolengo, Italy) was applied.

The distraction process started at 7 to 10 days after the surgery, at a rate of 1 mm per day. The endpoint of distraction included neutral ulnar variance for the skeletally mature patients, positive ulnar variance of no more than 5 mm for skeletally immature patients, and reduction of the radial head for patients with preoperative subluxation or dislocation. During the distraction process, we maintained forearm pronation and supination at least 50° each. If either the pronation or supination was less than 50° before surgery, the distraction proceeded only if there was no decline from preoperative range of motion. At the end of lengthening, the fixator was kept in place until bone consolidation.

Range of motion was recorded pre-operatively and at final follow-up. We measured pronation and supination at the most recent follow-up as the postoperative clinical outcome. The degree of lengthening was measured in centimeter. The length of external fixation time (EFT) was recorded, and the external fixation index (ETI) was calculated through dividing EFT by the degree of lengthening.

**Figure 1:**
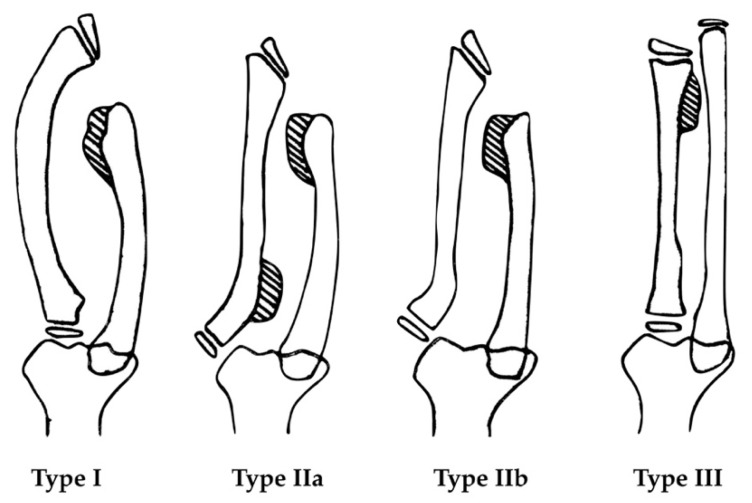
Masada classification [11]. **Type I**: The osteochondromas mainly occur at the distal ulna. The ulna is relatively shortened, and usually accompanied by radial bowing deformity. **Type IIa**: The radial head is dislocated with osteochondroma formation at the proximal radius. **Type IIb**: The radial head is dislocated without osteochondroma formation at the proximal radius. **Type III**: The radius is relatively shortened, and the osteochondroma forms at the distal radius.

**Figure 2:**
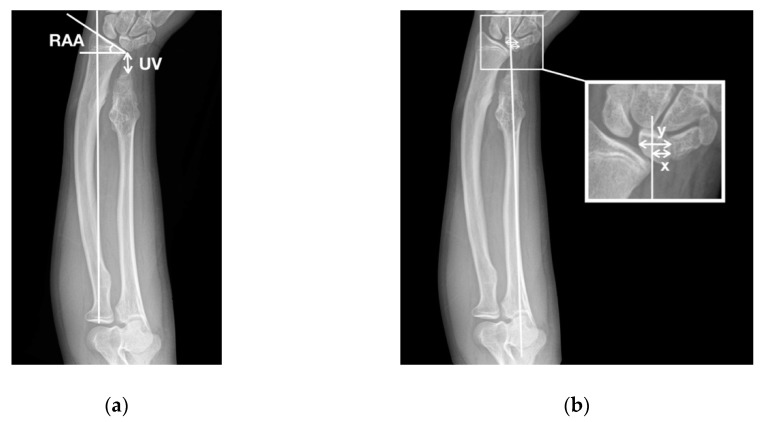
Radiographic parameters. (**a**) Ulnar variance (UV): the distance between the distal ulnar epiphysis and the ulnar side of the distal radial epiphysis. Radial articular angle (RAA): the angle defined between a line along the radial articular surface and a line perpendicular to the line drawn from the center of the radial head to the radial border of the distal radius. The normal value is 15–30°; (**b**) carpal slip (CS): the percentage of the lunate that falls on the ulnar side, with a reference line drawn from middle of the olecranon to the ulnar border of the radial epiphysis (*x*/*y* × 100%). The normal value is 50%.

Radiographic evaluation was conducted by plain radiographs in anterior–posterior and lateral views. The osteochondroma of the forearm was classified according to the Masada classification [11], as shown in Figure 1. The radiographic parameters included ulnar variance (UV), radial articular angle (RAA), and carpal slip (CS) by Fogel et al. [14], as shown in Figure 2.

We used SPSS version 22 (IBM Corp., Armonk, NY, USA) to conduct all statistical analyses and generate plots. To compare differences between the two groups, Fisher’s exact test and Mann–Whitney U test were used for dichotomous and continuous data, respectively. The pre- and post-operative values of radiographic and clinical measurements of each group were compared using Wilcoxon signed rank test. A *p* value < 0.05 was considered to be significant.

The study had been approved by the Institutional Review Board of the National Taiwan University Hospital (201901063RIN).

## 3. Results

The patient demographics are shown in Table 1. The elongated length was 21.3 ± 5.8 mm in the senior group and 22.5 ± 2.4 mm in the junior group, with an average postoperative follow-up of 38.7 ± 22 and 51.4 ± 18.6 months, respectively. In the senior group, the average EFT was 204.3 ± 63.7 days, and the EFI was 104.9 ± 53.6 days per cm. In the junior group, the average EFT was 159.0 ± 38.8 days, and the EFI was 51.4 ± 18.6 days per cm. During this period, non-union occurred in one patient in the senior group. It was treated by autologous iliac bone grafting and plate fixation. Delayed union was observed in one patient in the junior group, for which Kirschner wire fixation was applied after removal of the external fixator. No other complications were noted in this series.

**Figure 3:**
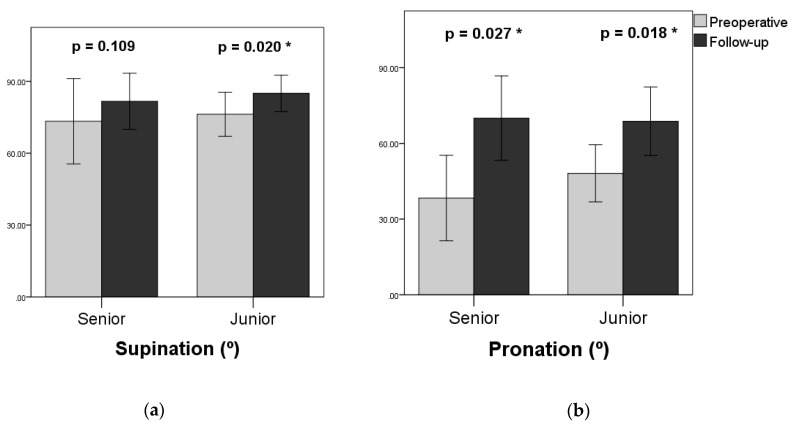
Comparisons between preoperative and follow-up range of motion. (**a**) Supination; (**b**) pronation. * *p* < 0.05.

The functional results are shown in Table 2 and Figure 3. In the senior group, supination increased from a preoperative average of 73.3° ± 16.2° to 81.7° ± 10.7° at the most recent follow-up, but did not reach statistical significance (*p* = 0.109). Pronation increased significantly from 38.3° ± 15.5° to 70.0° ± 15.3° (*p* = 0.027). In the junior group, supination increased from preoperative 76.3° ± 8.6° to 85.0° ± 7.1° (*p* = 0.020), and pronation increased from 48.1° ± 10.6° to 68.8° ± 12.7° (*p* = 0.018). The improvement in supination and pronation of the junior group reached statistical significance. One patient in the junior group who had a recurrence of negative ulnar variance ended with the same postoperative functional level as preoperative level.

**Figure 4:**
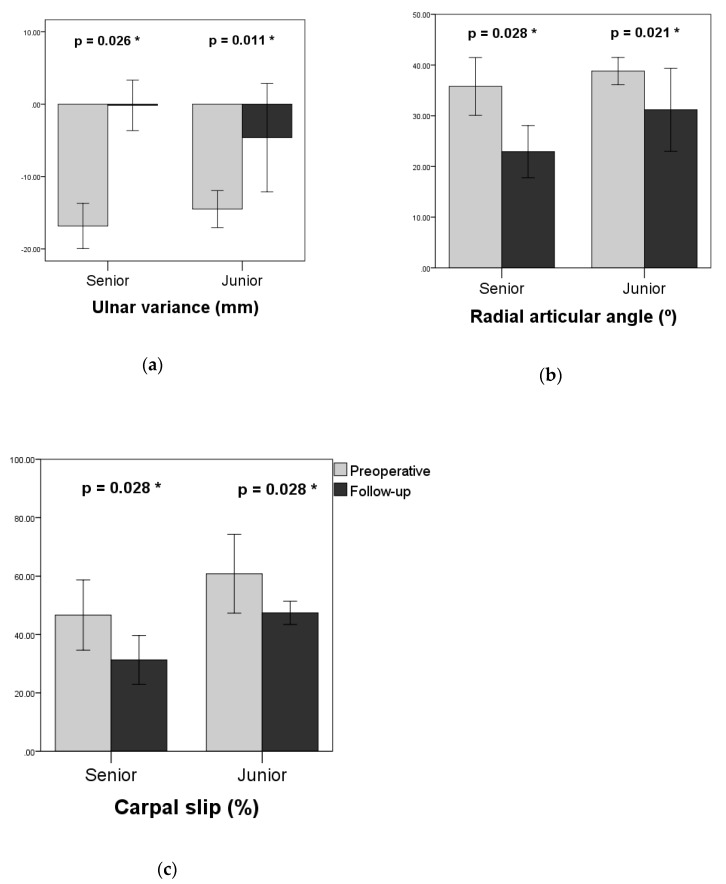
Comparisons between preoperative and follow-up radiographic measurements. (**a**) Ulnar variance; (**b**) radial articular angle; (**c**) carpal slip. * *p* < 0.05.

The radiographic results are shown in Table 3 and Figure 4. Significant improvement in radiographic parameters was observed in the senior group. The average negative UV was improved from −16.8 ± 2.9 mm to −0.2 ± 3.2 (*p* = 0.026). The average RAA decreased from 35.8° ± 5.2° to 22.9° ± 4.7° (*p* = 0.028). The average CS decreased from 46.7 ± 11.0% to 31.3 ± 7.6% (*p* = 0.028). In the junior group, the average negative UV was −14.5 ± 2.4, and improved to −4.6 ± 7.0 at the last follow-up (*p* = 0.011). The average RAA decreased from 38.8 ± 2.5° to 31.2 ± 7.7° (*p* = 0.021). The average CS decreased from 60.8 ± 12.3% to 47.4 ± 3.6% (and *p* = 0.028).

Spontaneous reduction of the radial head was observed after ulnar lengthening in the patient with type IIb deformity in the senior group. Residual radial head subluxation was still noted after lengthening in the patient with type IIa deformity in the senior group. A 9-year-old patient with type IIb deformity underwent repeated ulnar lengthening at 12 years due to recurrent ulnar shortening and radial head dislocation noted 18 months after the first lengthening. In this case, the radial head was successfully reduced after the second surgery.

**Figure 5:**
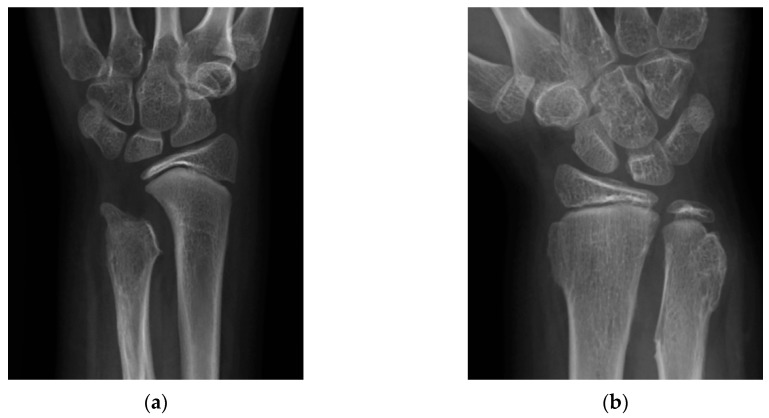
Radiographs of (**a**) a 10-year-old girl who underwent ulnar lengthening with recurrence two years later showed loss of the discoid nature of the distal ulnar physis; (**b**) a 9-year-old boy following ulnar lengthening at the age of three without recurrence showed smooth discoid nature of distal ulnar physis.

During the follow-up period, four patients in the junior group had a recurrence of negative ulnar variance. They underwent ulnar lengthening at age of 5, 9, 10, and 10 years, respectively. In reviewing the postoperative follow-up radiographs of these four patients, there was a loss of the discoid nature of the distal ulnar physis. This was in contrast to the patients without recurrence who had a relatively smooth and flat ulnar physis, as shown in Figure 5. One of the four patients had a deterioration of RAA. Only one patient with type IIb deformity underwent repeated ulnar lengthening.

## 4. Discussion

Ulnar lengthening by distraction osteogenesis has been a prevalent treatment option in the management of forearm deformities associated with MHE [10]. Although the forearm motion could be improved, simple excision of osteochondromas is not effective in controlling the progression of deformity [7,14].

A radial corrective osteotomy is commonly combined with ulnar lengthening to treat radial bowing deformity. Nevertheless, the necessity of this procedure in skeletally immature patients has not been discussed much in the current literature.

In skeletally immature patients, certain radial diaphysis malalignment may be accepted because of remodeling potential. In plastic deformation of forearms, Nimityongskul et al. recommended non-treatment for children under the age of five because anatomy and function will be self-corrected through remodeling. Correction of a severe deformity was suggested in children with angular of 20 degrees or more at the age of six to ten [12]. In forearm fractures, Tarmuzi et al. declared that 20 degrees of angulation could be managed conservatively in children younger than 10 years [13].

The effect of remodeling had been reported in studies of ulnar lengthening alone with significant improvements in the RAA compared to the preoperative measurements [15,16]. D’Ambrosi et al. performed a retrospective study in fifteen patients receiving gradual ulnar lengthening without radial angular correction at the age of 8 to 12. There were significant improvements in the RAA by the time they reached skeletal maturity [17]. In the present study, similar results were observed in the juniors (age ≤ 10 years) without concurrent radial corrective osteotomy. Only one patient had an increased RAA after ulnar lengthening at the age of five, which can be ascribed to the recurrence of radio-ulnar length discrepancy.

Results of the present study indicated good clinical outcomes in the junior patients without concurrent radial corrective osteotomy. Only one patient with recurrence of negative ulnar variance had the same postoperative functional level compared to the preoperative level. The rest of the patients, including the patient with an increased RAA, all had improvements in pronation and supination at the last follow-up visit. Compared with the older patients with relative skeletal maturity following dual procedures, ulnar lengthening alone in the younger patients can potentially restore radiologic anatomy and functions, providing comparable surgical outcomes in radiographic results and clinical parameters.

The optimal timing of surgical intervention is still debatable. Some authors suggested postponing lengthening to avoid recurrence [18,19], while other authors recommended early intervention [11,15,20]. Jo et al. reported the positive correlation between the amount of negative ulnar variance and both radial bowing and radial articular angle. The authors also observed a higher risk of radial head dislocation when the ratio of ulnar/radial length was less than 0.9 or the radial bowing was greater than 8.1% [21]. Early intervention can prevent or lower the progression of deformity and functional impairment. Most importantly, it can prevent the development of radial head dislocation which can manifest and contribute in a wide range of clinical symptoms [22]. In accordance with the current results, early intervention can enhance the remodeling potential of the younger patients and is in favor of spontaneous correction of the deformities, while skeletally mature patients often require additional corrective procedures.

Among the four patients with recurrence of a negative ulnar variance, only a 9-year-old patient underwent repeated ulnar lengthening. It is in agreement with the previous studies, suggesting that age is not a predictor of reoperation [23]. In addition, the patients with recurrence had a relatively defective appearance of the distal ulnar physis in the postoperative radiographs. In these cases, magnetic resonance imaging may be helpful to accurately evaluate the physeal integrity.

Many authors recommended initial overcorrection by 0.5 to 1.0 cm in skeletally immature patients to prevent recurrence [18,20,24,25]. On the contrary, Vogt et al. opposed preventive ulnar over lengthening in consideration of the ulnocarpal impaction syndrome and possibility in lack of compensation [16]. According to our protocol, decision to terminate the distraction was determined not only by the imaging studies. Rather, the forearm motion was continuously examined during the period of distraction to ensure that the minimal requirement for forearm function was retained. In patients with poor preoperative function, we ensured that there was at least no evidence in decline of function. The minimal requirement was set at 50° in both pronation and supination, according to a biomechanical study performed by Morrey et al. [26].

Spontaneous reduction of the radial head along with ulnar lengthening by distraction osteogenesis had been reported [24,27]. Along with the lengthening, the interosseous membrane between the ulna and radius can provide a distal pull of the radial head, allowing reduction of the head. In the present study, radial head reduction was achieved after ulnar lengthening in the two patients with no osteochondromas at the distal radius. The radial head remained subluxated in the patient with type IIa deformity. In this case, we did not continue the lengthening process to relocate the radial head after an overcorrection by 5 mm was reached and pronation was improved.

Tonogai et al. recommended dissection of the interosseous membrane when lengthening >20 mm in the proximal ulna to prevent radio-capitellar subluxation [28]. We instead performed ulnar osteotomy at the distal third of ulna on the patients without radial head dislocation and need of angular correction to provide more drag of the distal radioulnar articulation and less interference to the elbow joint. There were no treatment-induced radial head subluxation, dislocation or neurologic impairment observed in this series.

The limitations of our study include its retrospective nature and the relatively small sample size. As the angulation of the radial malformation could be in all directions, there could be discrepancies in our radiological evaluations which were based on two-dimensional images. Additionally, the surgical procedures employed in this series included ulnar lengthening, radial corrective osteotomy, and excisions of osteochondromas. It is therefore difficult to conclude that the clinical improvement was attributed to the correction of the negative ulnar variance, radial bowing, or tumor removal. Nonetheless, whether to perform the additional procedures to ulnar lengthening was determined by the protocol mentioned. The result of this study still provided valuable insights into gradual ulnar lengthening to treat the forearm deformity due to HME.

## 5. Conclusions

Ulnar lengthening by distraction osteogenesis is effective in improving both function and radiographic measurements of the forearm deformities in MHE patients. In senior patients older than 10 years, concurrent radial corrective osteotomy can be performed to correct the radial bowing deformity. In contrast, ulnar lengthening alone is an acceptable procedure in children younger than 10 years, considering the remodeling potential and functional improvement. Regarding HME patients in different age groups, our study provides a clinical implication to build an algorithm treatment in the future.

## Figures and Tables

**Table 1:** Demographic characteristics of patients.

	The Senior Group (*n* = 6) with Radial Correction	The Junior Group (*n* = 8) without Radial Correction	*p* Value
**Age (year)**	16.7 ± 4.4	8.1 ± 2.5	<0.01
Gender (Female/Male)	1/5	4/4	0.30
Side (Right/Left)	5/1	5/3	0.58
Masada Classification			
Type I	4	7	
Type IIa	1	0	
Type IIb	1	1	
Elongated length (mm)	21.3 ± 5.8	22.5 ± 2.4	0.43
EFT (day)	204.3 ± 63.7	159.0 ± 38.8	0.14
EFI (day/cm)	104.9 ± 53.6	72.0 ± 20.0	0.20
Follow-up (month)	38.7 ± 22	51.4 ± 18.6	0.12

EFT = external fixation time; EFI = external fixation index.

**Table 2:** Clinical outcomes.

	The Senior Group (*n* = 6)	The Junior Group (*n* = 8)	*p* Value
Supination			
Preoperative	73.3° ± 16.2°	76.3° ± 8.6°	0.95
Follow-up	81.7° ± 10.7°	85.0° ± 7.1°	0.61
Gained	8.3° ± 11.1°	8.8° ± 6.0°	0.59
Pronation			
Preoperative	38.3° ± 15.5°	48.1° ± 10.6°	0.29
Follow-up	70.0° ± 15.3°	68.8° ± 12.7°	0.89
Gained	31.7° ± 17.5°	20.6° ± 14.2°	0.30

**Table 3:** Radiographic outcomes.

	The Senior Group (*n* = 6)	The Junior Group (*n* = 8)	*p* Value
UV (mm)			
Preoperative	−16.8 ± 2.9	−14.5 ± 2.4	0.11
Follow-up	−0.2 ± 3.2	−4.6 ± 7.0	0.32
RAA (°)			
Preoperative	35.8 ± 5.2	38.8 ± 2.5	0.44
Follow-up	22.9 ± 4.7	31.2 ± 7.7	0.07
CS (%)			
Preoperative	46.7 ± 11.0	60.8 ± 12.3	0.11
Follow-up	31.3 ± 7.6	47.4 ± 3.6	0.01

UV = ulnar variance; RAA = radial articular angle; CS = carpal slip.

## References

[1] Sandell L.J. (2009). Multiple hereditary exostosis, EXT genes, and skeletal development. J. Bone Jt. Surg. Am..

[2] Chhina H., Davis J.C., Alvarez C.M. (2012). Health-related quality of life in people with hereditary multiple exostoses. J. Pediatr. Orthop..

[3] D’Ambrosi R., Ragone V., Caldarini C., Serra N., Usuelli F.G., Facchini R.M. (2017). The impact of hereditary multiple exostoses on quality of life, satisfaction, global health status, and pain. Arch. Orthop. Trauma Surg..

[4] D’Ambrosi R., Caldarini C., Ragone V., Facchini R.M. (2018). Effect of multiple hereditary exostoses on sports activity in children. J. Orthop..

[5] Schmale G.A., Conrad E.U., Raskind W.H. (1994). The natural history of hereditary multiple exostoses. J. Bone Jt. Surg. Am..

[6] Solomon L. (1961). Bone growth in diaphysial aclasis. J. Bone Jt. Surg. Br..

[7] Shin E.K., Jones N.F., Lawrence J.F. (2006). Treatment of multiple hereditary osteochondromas of the forearm in children: A study of surgical procedures. J. Bone Jt. Surg. Br..

[8] Rodgers W.B., Hall J.E. (1993). One-bone forearm as a salvage procedure for recalcitrant forearm deformity in hereditary multiple exostoses. J. Pediatr. Orthop..

[9] Kelly J.P., James M.A. (2016). Radiographic Outcomes of Hemiepiphyseal Stapling for Distal Radius Deformity Due to Multiple Hereditary Exostoses. J. Pediatr. Orthop..

[10] El-Sobky T.A., Samir S., Atiyya A.N., Mahmoud S., Aly A.S., Soliman R. (2018). Current paediatric orthopaedic practice in hereditary multiple osteochondromas of the forearm: A systematic review. SICOT J..

[11] Masada K., Tsuyuguchi Y., Kawai H., Kawabata H., Noguchi K., Ono K. (1989). Operations for forearm deformity caused by multiple osteochondromas. J. Bone Jt. Surg. Br..

[12] Nimityongskul P., Anderson L.D., Sri P. (1991). Plastic deformation of the forearm: A review and case reports. J. Trauma.

[13] Tarmuzi N.A., Abdullah S., Osman Z., Das S. (2009). Paediatric forearm fractures: Functional outcome of conservative treatment. Bratisl. Lek. Listy.

[14] Fogel G.R., McElfresh E.C., Peterson H.A., Wicklund P.T. (1984). Management of deformities of the forearm in multiple hereditary osteochondromas. J. Bone Jt. Surg. Am..

[15] Tang Z.W., Cao Y.L., Liu T., Chen T., Zhang X.S. (2013). Management of forearm deformities with ulnar shortening more than 15 mm caused by hereditary multiple osteochondromas. Eur. J. Orthop. Surg. Traumatol..

[16] Vogt B., Tretow H.L., Daniilidis K., Wacker S., Buller T.C., Henrichs M.P., Roedl R.W., Schiedel F. (2011). Reconstruction of forearm deformity by distraction osteogenesis in children with relative shortening of the ulna due to multiple cartilaginous exostosis. J. Pediatr. Orthop..

[17] D’Ambrosi R., Barbato A., Caldarini C., Biancardi E., Facchini R.M. (2016). Gradual ulnar lengthening in children with multiple exostoses and radial head dislocation: Results at skeletal maturity. J. Child. Orthop..

[18] Abe M., Shirai H., Okamoto M., Onomura T. (1996). Lengthening of the forearm by callus distraction. J. Hand Surg. Br..

[19] Ham J., Flipsen M., Koolen M., van der Zwan A., Mader K. (2016). Multiple osteochondromas (MO) in the forearm: A 12-year single-centre experience. Strateg. Trauma Limb Reconstr..

[20] Pritchett J.W. (1986). Lengthening the ulna in patients with hereditary multiple exostoses. J. Bone Jt. Surg. Br..

[21] Jo A.R., Jung S.T., Kim M.S., Oh C.S., Min B.J. (2017). An Evaluation of Forearm Deformities in Hereditary Multiple Exostoses: Factors Associated with Radial Head Dislocation and Comprehensive Classification. J. Hand Surg. Am..

[22] Stanton R.P., Hansen M.O. (1996). Function of the upper extremities in hereditary multiple exostoses. J. Bone Jt. Surg. Am..

[23] Refsland S., Kozin S.H., Zlotolow D.A. (2016). Ulnar Distraction Osteogenesis in the Treatment of Forearm Deformities in Children with Multiple Hereditary Exostoses. J. Hand Surg. Am..

[24] Matsubara H., Tsuchiya H., Sakurakichi K., Yamashiro T., Watanabe K., Tomita K. (2006). Correction and lengthening for deformities of the forearm in multiple cartilaginous exostoses. J. Orthop. Sci..

[25] Cho Y.J., Jung S.T. (2014). Gradual lengthening of the ulna in patients with multiple hereditary exostoses with a dislocated radial head. Yonsei Med. J..

[26] Morrey B.F., Askew L.J., Chao E.Y. (1981). A biomechanical study of normal functional elbow motion. J. Bone Jt. Surg. Am..

[27] Demir B., Gursu S., Ozturk K., Yildirim T., Konya M.N., Er T. (2011). Single-stage treatment of complete dislocation of radial head and forearm deformity using distraction osteogenesis in paediatric patients having multiple cartilaginous exostosis. Arch. Orthop. Trauma Surg..

[28] Tonogai I., Takahashi M., Tsutsui T., Goto T., Hamada D., Suzue N., Matsuura T., Yasui N., Sairyo K. (2015). Forearm lengthening by distraction osteogenesis: A report on 5 limbs in 3 cases. J. Med. Investig..

